# Caloric and video head impulse test dissociated results in dizzy patients

**DOI:** 10.3389/fneur.2022.1000318

**Published:** 2022-09-26

**Authors:** Sofia Waissbluth, Valeria Sepúlveda, Jai-Sen Leung, Javier Oyarzún

**Affiliations:** Department of Otolaryngology, Pontificia Universidad Católica de Chile, Santiago, Chile

**Keywords:** vertigo, dizziness, caloric test, video head impulse test, vestibular disease

## Abstract

**Introduction:**

We are now able to detect abnormalities for any semicircular canal with the use of the video head impulse test (vHIT). Prior to the vHIT, the gold standard for unilateral canal paresis of the lateral canal was considered the caloric test. Clinical cases where the caloric test and vHIT are discordant are not uncommon.

**Methods:**

Retrospective study. All consecutive cases of dizziness seen from 11/2020 to 12/2021 for which the patient underwent both caloric and vHIT tests performed within 10 days, were reviewed. Patients with discordant results were included. We evaluated the caloric response, vHIT gains for all canals and saccades, with and without gain abnormalities.

**Results:**

We included 74 cases of dizziness with dissociated results. The most common finding was a normal caloric response with abnormal vHIT results (60.8%); the main abnormal finding on vHIT was the presence of saccades. In this group, 37.7% of patients had normal gains and refixation saccades. In addition, the most found low gain was for the posterior canal. The main diagnosis in this group was vestibular migraine. For the group with unilateral caloric paresis and normal vHIT gain in the lateral canal, the main diagnosis was Ménière's disease.

**Discussion:**

The most common disorders with discordant results were Ménière's disease and vestibular migraine. The caloric test and vHIT are complementary and combining both tests provide greater clinical information. Further research is needed to understand refixation saccades with normal gains.

## Introduction

Recent evidence has shown that vestibular assessment with the caloric test and the video head impulse test (vHIT) can be discordant or dissociated (1). While both tests evaluate lateral semicircular canal function, they have important differences and limitations. The caloric test uses a non-physiological stimulus (≈0.006 Hz) to test the lateral canal and superior vestibular nerve while the vHIT tests the vestibulo-ocular reflex (VOR) at a high acceleration (≈2.5 Hz), considered a physiological stimulus of head rotation (2). It also provides information about all semicircular canals, superior and inferior vestibular nerve function, and overt and covert saccades (3). Prior to the implementation of the vHIT, the caloric test was considered the gold standard for testing lateral canal function. However, it is now understood that because these two tests evaluate at different frequencies and have different stimuli, they are complementary (4).

A recent study by Lee et al. reports that discordant results can be seen in approximately one out of every six patients with dizziness. However, this is considering that only horizontal vHITs were included in the analyses (5). They observed that the main diagnoses for patients with an abnormal caloric test but normal vHIT were Ménière's disease and vestibular neuritis/labyrinthitis. Similar findings were observed as a result of a systematic review and meta-analysis regarding discordant results in patients with chronic dizziness (4). As for an abnormal vHIT and normal caloric test, Lee et al. observed this finding in a variety of central and peripheral lesions (5).

The current study was undertaken to evaluate the etiology of patients with dizziness that underwent caloric testing and vHIT on the same day (or within 10 days), evaluate the patterns on vHIT for all semicircular canals, and the presence of refixation saccades.

## Methods

All consecutive cases of dizziness seen at the Pontificia Universidad Católica de Chile healthcare center from November 2020 until December 2021 for which the patient underwent both the caloric and vHIT tests, were reviewed. Inclusion criteria consisted of cases for which both tests were performed on the same day, or within 10 days. Cases were excluded if they had incomplete charts or if the tests were performed at another health care center. During this time period, 166 cases had both tests performed, however, 46 were excluded because the time period between both tests exceeded 10 days. A total of 120 cases were analyzed, of which, 46 were eliminated as both tests were concordant, hence, we finally included 74 cases of dizziness with dissociated results. This study was approved by the local Ethics Committee of the Pontificia Universidad Católica de Chile.

Bithermal caloric testing was performed with caloric stimuli consisting of alternate binaural irrigations with an air irrigator, with cold and hot temperatures (24 and 48°C) for 1 min. Nystagmus was recorded with videonystagmography (VisualEyes™ 525, Interacoustics) and peak slow-phase velocity was documented. Canal paresis was defined as a difference of ≥25% between both sides and was calculated using Jongkees' formula (6).

For vHIT testing, the right eye was recorded, and all three canals were evaluated (Otometrics ICS^®^ Impulse). During testing, subjects were fitted with the goggles, seated and asked to look at an eye-level target on the wall which is at a 1-meter distance. Following calibration, the examiner standing behind the patient, placed their hands on the participant's head and performed repeated head impulses which were randomized, in velocity and direction, in the plane of the tested semicircular canal. Head impulses (150–300°/s) were continued until 20 head impulses were adequate (artifact-free) for each tested canal. All head impulses were completed by experienced practitioners. Parameters of abnormality were as follows: lateral canal VOR gain < 0.8; vertical canal VOR gain < 0.7; and/or presence of corrective saccades (covert and/or overt) in any canal. The gain was calculated as the ratio of the area under the eye velocity and head velocity curve.

Results were considered discordant when: (1) normal caloric test and abnormal vHIT results (low gain in any canal and/or saccades in any canal), (2) unilateral canal paresis on caloric test with normal gain for the lateral canal on vHIT, and low gains for the posterior or anterior canals and/or ipsilateral and contralateral saccades, and (3) bilateral canal paresis on caloric test with normal gains for the lateral canals bilaterally on vHIT, and low gains for the posterior or anterior canals and/or saccades.

Depending on the history and clinical presentation, other tests were performed when considered necessary such as pure-tone audiometry, vestibular evoked myogenic potentials and brain MRI with or without posterior fossa protocol.

## Results

**Figure 1:**
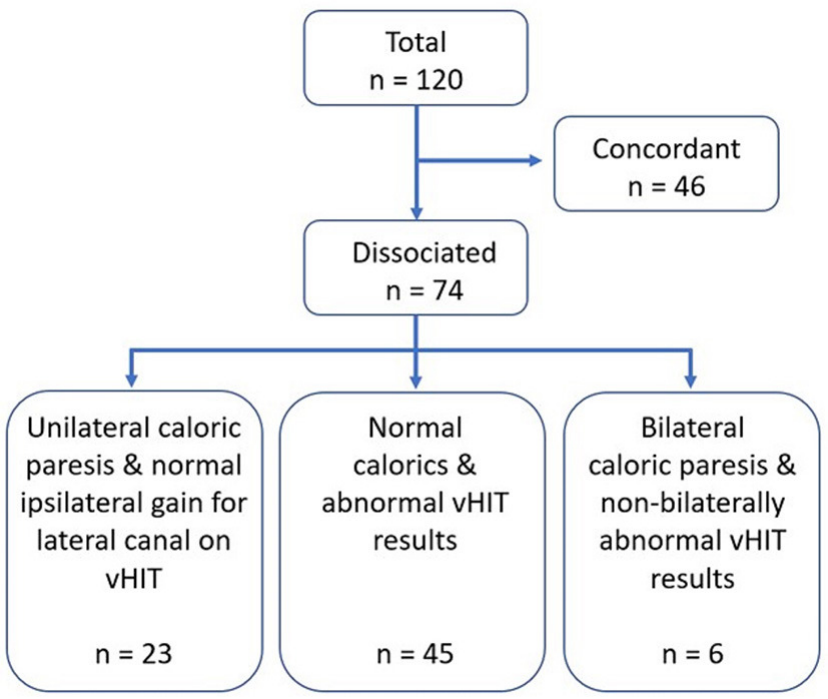
Flowchart of study participants.

Seventy-four cases of dizziness with dissociated results were evaluated (Figure 1). Patients were 58.6 ± 15.5 years on average, median of 59 years and range 10–85 years; 75.7% were women. The most common finding was presenting normal caloric testing with abnormal vHIT results (*n* = 45, 60.8%) followed by unilateral caloric paresis and normal vHIT gains for the lateral canal (*n* = 23). Six cases had bilateral canal paresis on caloric testing yet did not show abnormal bilateral vHIT gains for the lateral canals.

**Figure 2:**
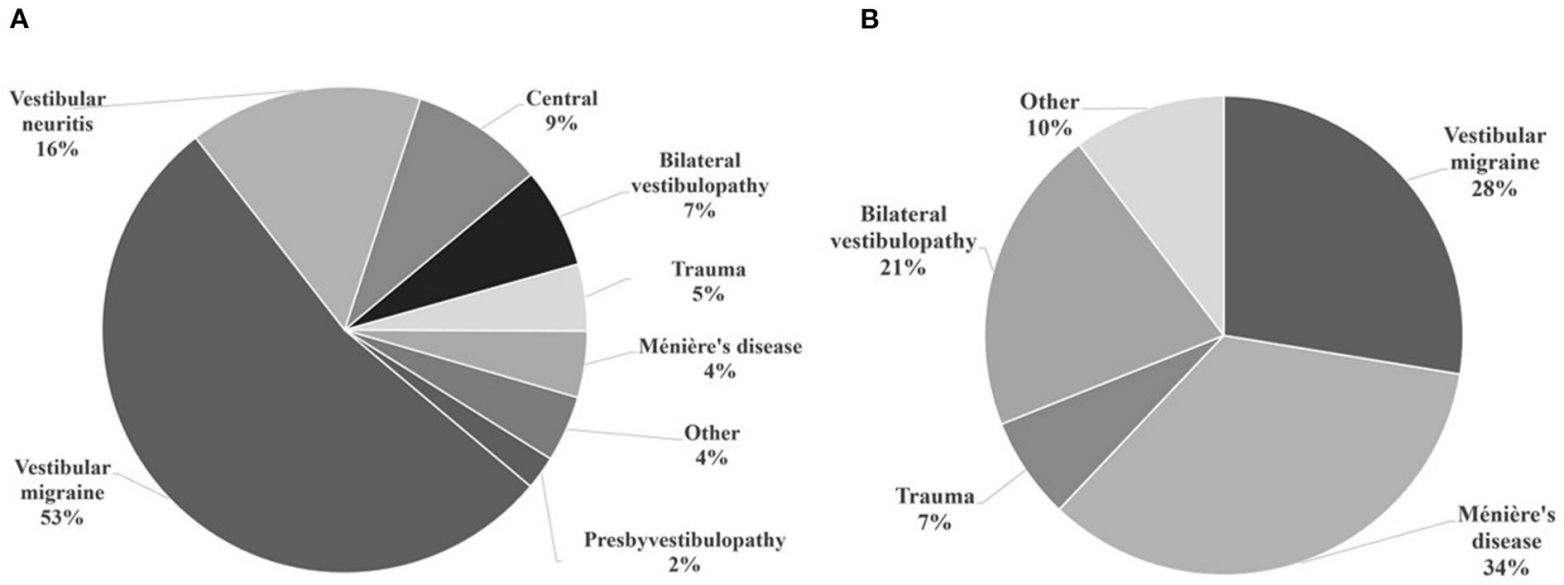
Otoneurological diagnoses of the included participants. **(A)** Normal caloric test and abnormal vHIT results. Common diagnoses in this category were vestibular migraine and vestibular neuritis (acute unilateral vestibulopathy). **(B)** Caloric paresis with dissociated vHIT findings. Common diagnoses in this category were Ménière's disease and vestibular migraine.

For the group with normal caloric testing with abnormal vHIT results, average gains for the lateral, posterior and anterior canals on the right side were: 0.93 ± 0.13, 0.74 ± 0.15 and 0.84 ± 0.16, and on the left side: 0.85 ± 0.14, 0.72 ± 0.19, and 0.78 ± 0.11, respectively. Overall, 71% of cases showed a low gain in any of the six canals, and 100% exhibited saccades in any canal (Table 1). The most commonly found low gain was for the posterior canal (56%), followed by the lateral canal (40%). Also, the most commonly found saccades were for the posterior canal (56%) followed by the lateral canal (40%). Although the percentages of low gains and saccades are coincidentally the same for the lateral and posterior canals, the saccades do not, however, necessarily correspond with the canal with low gain. When assessing per canal, we observed that 37.7% of canals had normal gains and refixation saccades, 37.7% had low gains with refixation saccades and 24.4% had low gains without any saccades. In this group, the most common diagnoses were vestibular migraine (53%) followed by vestibular neuritis (16%) (Figure 2A). No patient in this category had a low gain in an anterior canal and only four cases exhibited saccades for that canal.

**Table 1:** Normal caloric test and abnormal vHIT results.

**Normal caloric test (*****n*** **=** **45)**		
**vHIT low gains (*****n*** **=** **32)**		
In any canal	32	71%
Lateral canal low gain	18	40%
Lateral canal low gain—bilateral	5/18	
Posterior canal low gain	25	56%
Posterior canal low gain—bilateral	12 / 25	
Anterior canal low gain	0	0%
**vHIT saccades (*****n*** **=** **45)**		
In any canal	45	100%
Lateral canal	18	40%
Posterior canal	25	56%
Anterior canal	4	9%

For the group with caloric paresis and dissociated findings on vHIT (Table 2), unilateral canal paresis was observed in 23 cases and bilateral paresis in six cases. Average caloric paresis was 36.7 ± 12.9%. For the unilateral canal paresis cases, ipsilateral and contralateral saccades for the lateral canal was the most common finding on vHIT. Only three cases had a low gain for the posterior canal on the same side. This last finding was also observed for bilateral paresis cases (*n* = 6) where posterior canal low gain, ipsi- or contralaterally, were observed (*n* = 4). In this group, the most common diagnoses were Ménière's disease (34%) followed by vestibular migraine (28%) (Figure 2B).

**Table 2:** Caloric paresis and dissociated findings on vHIT.

**Unilateral caloric paresis (*****n*** **=** **23)**		
**vHIT ipsilateral low gain (*****n*** **=** **3)**		
Lateral canal	0	0%
Posterior canal	3	13%
Anterior canal	0	0%
**vHIT ipsilateral saccades (*****n*** **=** **9)**		
Lateral canal only	6	26%
Lateral and posterior canals	3	13%
Anterior canal	0	0%
**vHIT contralateral saccades (*****n*** **=** **8)**		
Lateral canal only	6	26%
Posterior canal only	2	9%
Anterior canal	0	0%
**Bilateral caloric paresis (*****n*** **=** **6)**		
vHIT lateral canal low gain - bilateral	0	0%
vHIT lateral canal low gain - unilateral	2	33%
vHIT posterior canal low gain - unilateral or bilateral	4	67%
vHIT anterior canal low gain	0	0%
Saccades in any canal	5	83%

A few other aspects that were also evaluated was the presence of spontaneous nystagmus and directional preponderance on caloric testing. Fifteen patients had spontaneous nystagmus; five had Ménière's disease, five had vestibular migraine, three had a central etiology, one had vestibular neuritis and one had a labyrinthine infarction. Eight of these patients had caloric paresis and dissociated findings on vHIT, six had spontaneous horizontal nystagmus beating away from the affected side, but two had nystagmus toward the affected side and these patients had Ménière's disease. Seven had normal caloric testing with abnormal vHIT results, with a myriad of combinations; low gains and/or saccades, unilateral or bilateral, involving any canal. Only two cases had a directional preponderance on caloric testing (≥40%), one had a lateral semicircular canal dysplasia and one had vestibular migraine.

Because the most common diagnosis in this cohort was Ménière's disease, we decided to look into enhanced eye velocity on vHIT testing since prior evidence has suggested that this may be a result of endolymphatic hydrops. Twenty-six patients had a VOR >1 for the lateral canal (average gain: 1.07 ± 0.06). Interestingly, all were for the rightward head impulse, and of these, six were bilateral. This directional bias has been previously described for area under the curve gains (i.e., Otometrics^®^) with a consistent directional bias, with gains being larger in the ipsilateral direction of the eye used to measure gain (right eye in this case) (7). Thirteen had vestibular migraine and five had Ménière's disease. We did not encounter any cases with VOR gains >1.29, a recently described cutoff value based on gains obtained from healthy subjects (8).

As various patients had normal gains with saccades, we decided to analyze this group of patients as well. Overall, 22 patients had normal gains on vHIT and saccades in at least one canal; 14 patients had a normal caloric response and eight had unilateral caloric paresis. Ninety percent had saccades in the lateral canal. This most common diagnoses for these patients was vestibular migraine (54.5%), followed by vestibular neuritis (22.7%).

## Discussion

With the introduction of the vHIT in clinical practice, we are now able to detect abnormalities for any semicircular canal and clearly view overt and covert saccades. Prior to the vHIT, the gold standard for unilateral canal paresis of the lateral canal was considered the caloric test, as well as rotatory chair, mostly for bilateral paresis. As we now understand the intricacies and importance of testing different frequencies, we have come across clinical cases where the caloric and vHIT are not concordant, and it is not uncommon to have one of the two tests with abnormalities while the other is normal. With our criteria for discordant results, we observed that 74 out of 120 clinical cases had some degree of discordance, being the most frequent presentation that of a normal caloric test with abnormal vHIT results (60.8%), and that within this group, all patients had saccades in at least one canal (Table 1). Recently, Li et al. also evaluated discordance rates in 65 patients, and they observed that 55 patients had caloric weakness with a normal horizontal vHIT. However, they also observed that 36.4% had corrective saccades on the abnormal caloric side (9). Their conclusions are based on the horizontal canals, and therefore differ from ours, since they report that an abnormal caloric test with negative horizontal vHIT was mostly found.

The most common otoneurological disorders with discordant results were Ménière's disease and vestibular migraine. All these cases were diagnosed based on the consensus documents of the Classification Committee of the Bárány Society (10, 11). These happen to also be the most common causes of spontaneous episodic vestibular syndrome. We observed that the most common diagnosis for unilateral canal paresis with dissociated vHIT results was Ménière's disease; this is consistent with recently published data (12–15). It appears that the caloric test is more sensitive for detecting vestibular abnormalities in Ménière's disease, however, concomitant use of the vHIT enables the clinician to detect abnormal gains in vertical canals (16), and also demonstrates the presence of overt and covert saccades. This is of interest because both tests provide different types of information. It is believed that the caloric test in Ménière's disease is abnormal as a result of the physical enlargement of the membranous labyrinth by endolymphatic hydrops with a resulting localized convective flow that dissipates hydrostatic pressure across the cupula, and causes the cupular and hair cell deflection to be reduced (1). This is a non-physiological stimulus to test the lateral canal and superior vestibular nerve and does not provide information about saccades. On the other hand, the vHIT tests the VOR at a high acceleration, considered a physiological stimulus (2). Considering the hydrostatic temperature dissipation hypothesis previously mentioned, an increase in the semicircular duct diameter would have little effect on the response of the canal to angular acceleration stimulation (1, 3). This is compatible with the current findings and previously published data regarding caloric test and vHIT discrepancies in Ménière's disease.

While 34% of cases in the unilateral canal paresis with dissociated vHIT results group had Ménière's disease, 28% had vestibular migraine. This was also the most common diagnosis in the normal caloric test and abnormal vHIT results group. While vestibular migraine is a clinical diagnosis, a variety of examination findings and vestibular test abnormalities, both ictal and interictal, have been reported (17, 18). Most studies evaluating both test results in vestibular migraine report greater hypofunction on caloric testing vs. vHIT abnormalities (14, 19–22), and discordant results have been observed (14, 15, 21). However, some authors considered vHIT as abnormal when there was a low canal gain and saccades (22), or reported the vHIT as abnormal when the gain was low for the lateral canal (14). Mahringer and Rambold evaluated patients with caloric paresis to assess discordance with vHIT results (21). They analyzed cases with a pathological unilateral weakness on caloric testing (absolute value ≥ 25%). Of the 18 patients with vestibular migraine, two had a pathological unilateral vHIT. On the other hand, Janiak-Kiszka et al. recently evaluated 33 patients with vestibular migraine and none had caloric paresis (23). Potential explanations for our results could be that (1) The patients included were seen at their initial presentation and perhaps with a longer follow-up, results would vary as it has been suggested that vestibulo-cochlear dysfunction progresses slowly in some patients with vestibular migraine (24), (2) We only included patients with discordant results, and (3) The sample size is too small to take into account the great variability seen in vestibular tests for vestibular migraine.

Yilmaz et al. reported that 18% of patients with vestibular migraine with normal caloric testing had abnormal vHIT results; and so, this is not a rare finding (14). Interestingly, the main abnormal finding on vHIT when the caloric test was normal, was the presence of saccades. In this group, we observed that 37.7% had normal gains and refixation saccades. ElSherif et al. observed that 18.8% of patients with vestibular migraine had saccades with normal VOR gains (25), while Yollu et al. report that 52.4% of VM patients had saccades, and 28% had an abnormal gain for any canal (26). In our cohort, vestibular migraine was the most frequent diagnosis with normal gains with saccades in at least one canal, followed by vestibular neuritis. For the latter, evidence has shown that vHIT gains tend to recover while saccades can be detected at follow-up when gains have already recovered. Hence, it is not uncommon to observe saccades in the lateral canal in a patient who experienced vestibular neuritis in the months prior (27).

The pathophysiology of vestibular migraine is still unclear, but recent advances in structural and functional imaging have shown altered connectivity patterns in these patients (28–30). Structurally, it has been described that patients with vestibular migraine have a selective gray matter volume increase in the frontal and occipital regions, as well as of the left thalamus (31). When stimulated with ear irrigation, a significant increase in thalamic activation has been observed with functional MRI (fMRI) (30). Other findings on fMRI have been enhanced functional connectivity between the auditory network and the salience network and decreased functional connectivity in the bilateral medial cingulate gyrus and paracingulate gyrus within the sensorimotor network (28). The debate is ongoing as to whether vestibular migraine is a functional central vestibular disease, with or without structural changes, or whether the origin is mostly central or peripheral. Findings suggest that there may be an increased sensitivity to vestibular sensory processing (28), and an abnormal brain sensitization leading to altered multimodal sensory integration and processing cortical areas in these patients (31). Also, there is evidence for the existence of an interconnected trigemino-vestibular neuro-circuitry, which includes connections between the trigeminal system, the vestibular nuclei and the vestibulocerebellum (32). Interestingly, a small, proof-of-concept study, has shown that external trigeminal nerve stimulation produces some relief of vestibular migraine attacks (32).

The reason for saccades with normal gain values in vestibular migraine remains to be determined. Interestingly, Pérez-Fernández and Eza-Nuñez evaluated patients with dizziness with vHIT in order to evaluate whether refixation saccades alone had a localizing value when lateral canal gain was normal. Of the 36 patients included in their study, 29 had Ménière's disease and overall, the caloric test was abnormal in 60% (33).

Our study has limitations. It is a retrospective study which can have a selection bias. Our sample size is considered small because the main diagnoses were episodic vestibular syndromes that vary based on ictal/interictal presentations or attacks, hence, a larger sample would provide further information. Our subsample of bilateral vestibulopathy was too small to assess discordance. Also, we do not have access to, or have results for, rotary chair testing, which would have provided further details regarding lateral canal function.

## Data availability statement

The raw data supporting the conclusions of this article will be made available by the authors, without undue reservation.

## Ethics statement

The studies involving human participants were reviewed and approved by Ethics Committee of the Pontificia Universidad Católica de Chile. Written informed consent for participation was not required for this study in accordance with the national legislation and the institutional requirements.

## Author contributions

SW designed the study and wrote the first draft of the manuscript. SW and VS evaluated and collected data and performed data analyses. J-SL and JO collected data. All authors contributed to the article and approved the submitted version.

## Funding

This work was supported by the Fondo Nacional de Desarrollo Científico y Tecnológico (FONDECYT-ANID) grant 11201142 for SW.

## Conflict of interest

The authors declare that the research was conducted in the absence of any commercial or financial relationships that could be construed as a potential conflict of interest.

## Publisher's note

All claims expressed in this article are solely those of the authors and do not necessarily represent those of their affiliated organizations, or those of the publisher, the editors and the reviewers. Any product that may be evaluated in this article, or claim that may be made by its manufacturer, is not guaranteed or endorsed by the publisher.

## Supplementary material

Supplementary Figure 1 vHIT result for a patient with Ménière's disease. The patient had a caloric paresis of the right side (30%), however, the vHIT showed a decreased gain for the left lateral canal (0.74) and covert saccades for the lateral canals.

Supplementary Figure 2 vHIT result for a patient with vestibular neuritis. The patient had a normal caloric response, however, the vHIT showed decreased gains for the left lateral (0.67) and posterior (0.66) canals, and overt saccades for the left lateral canal.
