# Supplementary figures and images for: Caloric and video head impulse test dissociated results in dizzy patients
Source: Front Neurol. 2022 Sep 26;13:1000318. doi: 10.3389/fneur.2022.1000318 (PMC9548977; doi:10.3389/fneur.2022.1000318)

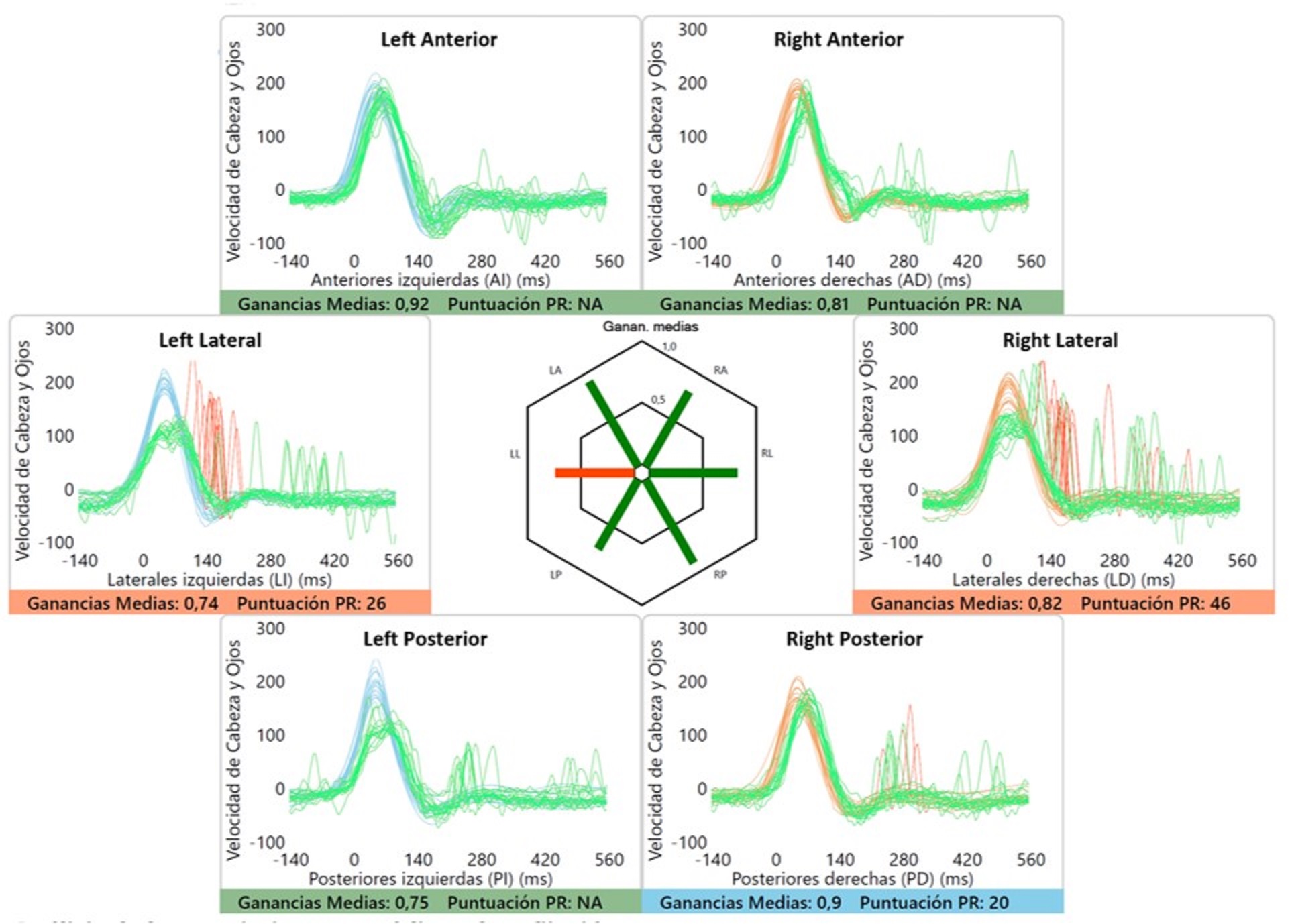

Supplement: Supplementary Figure 1 — vHIT result for a patient with Ménière's disease. The patient had a caloric paresis of the right side (30%), however, the vHIT showed a decreased gain for the left lateral canal (0.74) and covert saccades for the lateral canals. [file Image_1.JPEG]

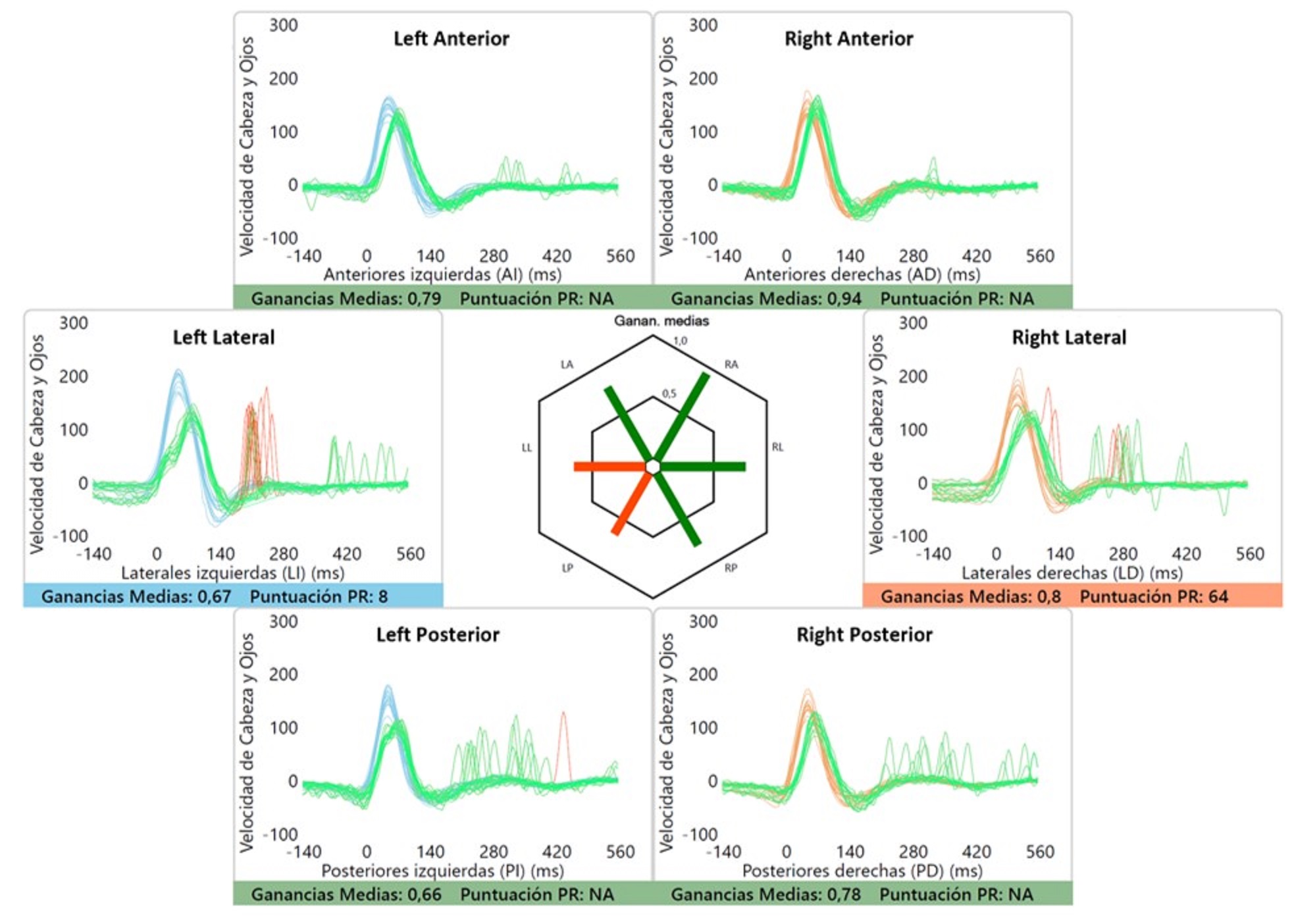

Supplement: Supplementary Figure 2 — vHIT result for a patient with vestibular neuritis. The patient had a normal caloric response, however, the vHIT showed decreased gains for the left lateral (0.67) and posterior (0.66) canals, and overt saccades for the left lateral canal. [file Image_2.JPEG]
